# Modeling mitigation of influenza epidemics by baloxavir

**DOI:** 10.1038/s41467-020-16585-y

**Published:** 2020-06-02

**Authors:** Zhanwei Du, Ciara Nugent, Alison P. Galvani, Robert M. Krug, Lauren Ancel Meyers

**Affiliations:** 1 Department of Integrative Biology, University of Texas at Austin, Austin, TX USA; 2 Department of Statistics and Data Science, University of Texas at Austin, Austin, TX USA; 3 Center for Infectious Disease Modeling and Analysis, Yale School of Public Health, New Haven, CN USA; 4 Department of Molecular Biosciences, John Ring LaMontagne Center for Infectious Disease, Institute for Cellular and Molecular Biology, University of Texas at Austin, Austin, TX USA; 5 Santa Fe Institute, Santa Fe, NM USA

**Keywords:** Diseases, Health care

## Abstract

Influenza viruses annually kill 290,000–650,000 people worldwide. Antivirals can reduce death tolls. Baloxavir, the recently approved influenza antiviral, inhibits initiation of viral mRNA synthesis, whereas oseltamivir, an older drug, inhibits release of virus progeny. Baloxavir blocks virus replication more rapidly and completely than oseltamivir, reducing the duration of infectiousness. Hence, early baloxavir treatment may indirectly prevent transmission. Here, we estimate impacts of ramping up and accelerating baloxavir treatment on population-level incidence using a new model that links viral load dynamics from clinical trial data to between-host transmission. We estimate that ~22 million infections and >6,000 deaths would have been averted in the 2017–2018 epidemic season by administering baloxavir to 30% of infected cases within 48 h after symptom onset. Treatment within 24 h would almost double the impact. Consequently, scaling up early baloxavir treatment would substantially reduce influenza morbidity and mortality every year. The development of antivirals against the SARS-CoV2 virus that function like baloxavir might similarly curtail transmission and save lives.

## Abstract (web summary)

Here, the authors implement a mathematical model that describes how Baloxavir antiviral-induced inhibition of influenza virus replication in infected individuals affects the spread of the virus during epidemics, suggesting that both the scaling up and acceleration of treatment would avert substantial influenza morbidity and mortality every year.

## Introduction

Influenza A and B viruses cause a highly contagious respiratory disease in humans that kills 290,000–650,000 people worldwide every year^1^. Vaccination is the primary means for controlling influenza transmission but is hampered by the variable efficacy and incomplete population coverage of annual vaccines, and thus is not yet sufficient for preventing large annual epidemics. Antiviral medications can shorten the duration of symptoms and reduce the likelihood of severe outcomes when administered to infected individuals shortly after they develop symptoms. Prior to 2018, the only approved influenza antivirals were viral neuraminidase inhibitors^2,3^. Of these, only oseltamivir (Tamiflu) can be taken orally, thereby facilitating its widespread usage. Oseltamivir inhibits the release of progeny virus from the cell surface, which is the last step in the production of infectious virus. Multiple oseltamivir treatments over 5 consecutive days are required to fully arrest virus production.

In 2018, a new oral antiviral, baloxavir (Xofluza), was approved in the United States for use in adults^4^. Baloxavir inhibits an early step in virus replication, the initiation of viral mRNA synthesis^5–7^. This initiation step requires cap-snatching, a mechanism in which the viral polymerase binds to the cap structure (m^7^GpppNm) at the 5’ ends of pre-mRNAs, the nuclear RNA precursors to cellular mRNAs, and then the endonuclease enzyme in the polymerase itself cleaves the pre-mRNAs at a position 10–14 bases downstream from the cap to generate the capped RNA fragments that serve as primers to initiate viral mRNA synthesis. Because baloxavir almost completely inhibits the cap-dependent endonuclease, little or no initiation of viral mRNA synthesis occurs, and little or no virus is produced. Consequently, as predicted, baloxavir treatment of infected patients almost totally inhibits virus production rapidly, within 24 h^8^. For this reason, only a single dose of baloxavir is needed to block virus production and shorten symptoms.

In addition to reducing the duration of symptoms, influenza antivirals can reduce infectiousness by shortening the period of virus shedding. In fact, because baloxavir treatment rapidly inhibits virus replication, virus shedding is shortened by 2–3 days. Consequently, widespread baloxavir treatment is predicted to substantially reduce population-level incidence, analogous to the herd effect attributed to vaccines^9^. Here, we estimate the impact of increasing baloxavir treatment coverage and varying times of treatment on population-level incidence using both clinical results and a hierarchical mathematical model that links within-host dynamics of viral load to between-host transmission. Our results indicate that scaling up and accelerating baloxavir treatment would substantially reduce influenza morbidity and mortality every year.

## Results and discussion

### Impact of antiviral treatment on the cell-to-cell proliferation of influenza

**Fig. 1:**
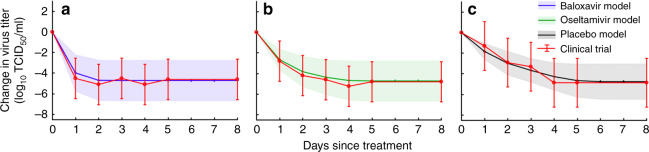
Changes in viral load following antiviral treatment. The estimated means and standard deviations in the change in virus titer from the fitted within-host model track the empirical observations^8^ among patients treated with **a** baloxavir (427 patients), **b** oseltamivir (377 patients), or **c** placebo (210 patients). Day zero corresponds to the time of the first dose.

Our within-host model assumes that infected patients have an initial load of drug-sensitive virus that increases via replication and decreases via immune response and antiviral treatment^10,11^ (Supplementary Fig. 1). We estimated the efficacy with which oseltamivir and baloxavir inhibit viral replication by fitting the model to the results of a recent clinical trial^8^ that measured the viral loads of 1014 influenza virus-infected patients after treatment with oseltamivir, baloxavir, or a placebo (Table 1). Our model produces viral titer estimates similar to the clinical data, and, like the clinical data, shows that baloxavir inhibits influenza virus replication more effectively than oseltamivir (Fig. 1). Within 1 day of initiating baloxavir or oseltamivir treatment, virus load decreases by an estimated 84% or 56%, respectively, compared with an expected reduction in untreated cases of 39%. The observed differences in the time between symptom onset and the initiation of treatment for patients in the clinical trial accounts for most of the observed variability in virus replication (Fig. 1, standard deviations). We used the fitted model to predict the effectiveness of drug treatment scenarios beyond those tested in the clinical trial, including the initiation of baloxavir or oseltamivir regimens at different times after symptom onset (Supplementary Fig. 3).

**Table 1:** Summary of key parameter estimates from fitting within-host and between-host models to data.

Parameter	Median	95% CI lower	95% CI upper
Antiviral efficacy $\epsilon$ for baloxavir	0.9997	0.9996	0.9999
Antiviral efficacy $\epsilon$ for oseltamivir	0.89	0.88	0.90
Initial sensitive viral load *V*_*0*_ (TCID_50_/ml)	258.2	3.3^a^	2268.9^a^
Basic reproduction number *R*_0_ in 2016–2017 season	1.09	1.06	1.11
Basic reproduction number *R*_0_ in 2017–2018 season	1.15	1.12	1.17
Basic reproduction number *R*_0_ in 2018–2019 season	1.10	1.08	1.13
Baseline distribution of treatment initiation time, *G*_0–48_ (hours after symptom onset, truncated at 48 h)	*G*(4.0, 6.3)		
Accelerated distribution of treatment initiation times, *G*_0–24_ (hours after symptom onset, truncated at 24 h)	*G*(4.0, 6.3)/2		
Delayed distribution of treatment initiation times, *G*_24–48_ (hours after symptom onset, compressed to 24–48 h window)	*G*(4.0, 6.3)/2 + 24		
Distribution of time lag between infection and symptom onset, *L* (hours)	24^a^*L*(0.37,0.41)		

^a^For estimates derived by simulated annealing, we provide 95 percentile range rather than confidence intervals.

Viral replication and antiviral efficacy are estimated via simulated annealing^46^ and approximate Bayesian computation^38,39,47^ fitting of deterministic within-host model to clinical trial data;^8^ season-specific transmission rates are estimated via approximate Bayesian computation^38,39^ fitting of stochastic population-level influenza transmission model (Supplementary Section 1) to US seasonal influenza incidence data^12^. Parameters for distributions of time between infection and symptom onset (lognormal) and from symptom onset to treatment (gamma) were estimated by the interior-point algorithm fitting of clinical trial data^8^. The key parameter estimates of within-host model and between-host model are summarized here, whereas others are in Supplementary Tables 2 and 3.

### Impact of baloxavir treatment on the transmission dynamics of influenza

**Fig. 2:**
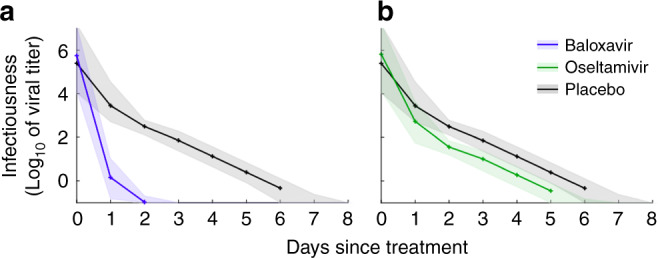
Early antiviral treatment reduces the infectiousness of influenza virus. Reduction of infectiousness by treatment with **a** baloxavir or **b** oseltamivir compared with treatment with placebo. Patients were assumed to be treated within 48 h of symptom onset, with the same scheduling as in the clinical trial^8^. Lines and shading indicate medians and interquartile ranges across 1014 stochastic simulations, corresponding to the sample sizes of 427, 377, and 210 for the baloxavir, oseltamivir, and placebo clinical trial groups, respectively.

We incorporated this viral replication model into a stochastic individual-based model of influenza transmission that tracks the daily evolution of infectiousness with disease progression. The infectiousness of a case at any given time depends on viral load, treatment status, and baseline transmission rates estimated from influenza surveillance data^12,13^ (Supplementary Table 2). Consistent with previous studies^14,15^, we assume a logarithmic relationship between viral load and infectiousness (Fig. 2). Unless otherwise specified, each course of treatment is initiated within the first 48 h of symptom onset, with the exact treatment times following the distribution reported in the recent clinical trial^8^ (Table 1). A day after initiating treatment with baloxavir or oseltamivir, the model projects that infectiousness is reduced by 95% or only 21%, respectively, relative to a comparable untreated patient (Fig. 2a). In addition, baloxavir-treated patients are likely to become non-infectious within two days, whereas oseltamivir-treated patients are predicted to remain infectious for ~4 or 5 days.

**Fig. 3:**
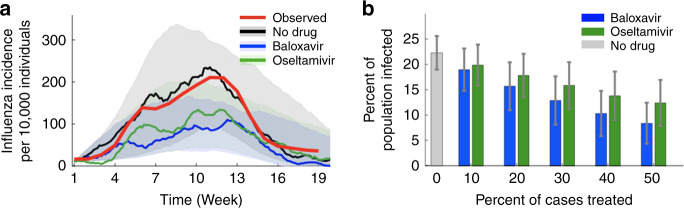
Mitigation of seasonal influenza with mass antiviral treatment. **a** Observed incidence of influenza virus (red) based on US surveillance data from the 2017–2018 influenza season compared with typical model simulations without any antiviral treatment (black) or with 30% of cases receiving baloxavir (blue) or oseltamivir (green) treatment. Lines indicate a moving 10-day average of incidence; shading corresponds to the 80% prediction interval across 1000 stochastic simulations. **b** Estimated total attack rates in simulated intervention scenarios ranging from no cases receiving antiviral treatment to 50% of cases treated. The heights of the columns and error bars show the median values and interquartile range, respectively, across *n* = 1000 independent stochastic simulations for each scenario. Each stochastic simulation assumes a population of 10,000 individuals, with within-host viral replication and between-host transmission parameters given in Table 1.

To project the population-level impacts of both scaling up and accelerating antiviral treatment, we fit our model to the 2017–2018 influenza epidemic in the United States, a severe epidemic which resulted in an estimated 63.3 million people infected, over 900,000 hospitalizations and more than 79,000 deaths^16^. In the absence of scaling up antiviral coverage, the timing and magnitude of the epidemiological trajectories projected by the model match the 2017–2018 seasonal epidemic (Fig. 3a). Treatment of 30% of infected cases with baloxavir or oseltamivir within 48 h after symptoms onset reduces the expected number of influenza infections throughout the virus season by 38% or 26%, respectively. We estimate the reduction in the number of overall infections at other treatment levels, ranging from 10% to 50% (Fig. 3a). As the percent of cases receiving antiviral treatment is increased, the estimated herd effect increases as reflected by a proportional decline in expected incidence. Baloxavir treatment is predicted to reduce the overall burden of influenza more than oseltamivir treatment across all treatment rates. If half of all cases are treated, baloxavir or oseltamivir are expected to reduce incidence by 58% or 39%, respectively. Similar herd effects are estimated for models that are fit to incidence data from the 2016–2017 and 2018–2019 influenza seasons in the United States (Supplementary Figs. 4 and 5). For each intervention scenario in the 2017–2018 season, we also calculated the basic reproduction number (*R*_0_), the average number of secondary infections generated by a typical infectious case at the outset of the epidemic (Supplementary Table 2). For example, treatment of 30% of cases with baloxavir would reduce *R*_0_ from a 2017–2018 baseline of ~1.15 (95% CI 1.12, 1.17) to ~1.08 (95% CI 1.05, 1.10).

**Fig. 4:**
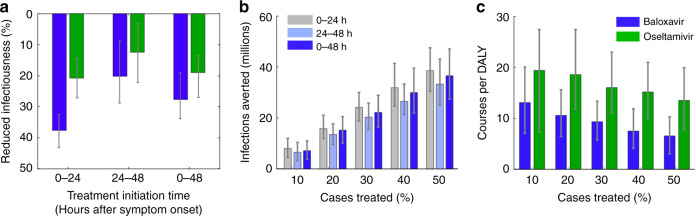
Public health impacts of expanding and accelerating antiviral treatment. **a** The estimated overall reduction in infectiousness in a treated individual resulting from antivirals, depending on the timing of their first dose after symptom onset. Values are areas between the log viral titer curves shown in Fig. 2 for untreated and treated cases. The 0–48 h treatment window assumes treatment times follow the distribution reported in the recent clinical trial^8^ (Table 1); the 0–24 and 24–48 h treatment windows use compressed versions of the 48 h empirical distribution. The heights of the columns and error bars indicate median values and interquartile ranges, respectively, across 100 stochastic simulations for each treatment window. **b** Total infections averted by treating the indicated percent of cases with baloxavir within 24 h, between 24 and 48 h, and within 48 h of symptom onset. For each treatment window, we compress the empirical 48-hour distribution of antiviral administration times. **c** The number of courses of treatment per DALY averted resulting from treating the indicated percent of cases with either baloxavir or oseltamivir within 48 h of symptom onset. For both graphs, the height and error bars indicate medians and interquartile ranges across *n* = 1000 independent pairs of simulations (baseline vs. treatment scenarios) of the fitted 2017–2018 model.

Using our model based on the 2017–2018 influenza season, we consider the population-level impacts of treatment initiation time within the 48 h period after symptom onset. Both the efficacy of baloxavir treatment and the increased benefit of baloxavir relative to oseltamivir are greatest in the first 24 h period (Fig. 4a). For a single infected individual, baloxavir treatment administered within the first 24 h period is expected to achieve nearly double the reduction in infectiousness (87%) than treatment administered within the second 24 h period. On a population level, baloxavir treatment within the first 24 h after symptoms onset results in a significantly greater reduction in total incidence than treatment within the second 24 h window following symptom onset (Fig. 4b). At the 30% and 50% case treatment rates, the early baloxavir treatment scenario is expected to avert 3.8 and 5.3 million infections more than the delayed treatment scenario, respectively. We also evaluated the distribution of treatment times reported in the baloxavir clinical trial:^8^ approximately equal numbers of patients treated in the 0–24 and 24–48 h time periods following symptom onset. This mixture is expected to reduce transmission to almost the same extent as accelerating all treatment to within 24 h of symptom onset (Fig. 4b). We restrict our analysis to treatment initiated within the initial 48 h window, given that later treatment will only negligibly impact incidence and that treatment within 48 h is clearly indicated^17,18^. In addition, treatment within 48 h is increasingly feasible with the expansion of telemedicine and online clinics (e.g., through the Xofluza website^19^ and insurance providers^20^).

Finally, we estimate influenza-associated mortality and morbidity averted by scaling up baloxavir or oseltamivir treatment (Fig. 4c). Specifically, we calculate the reduction in Disability-Adjusted Life Years (DALYs)^21^ between simulated 2017–2018 epidemics with and without scaling up antiviral treatment. For averted cases, we use DALY estimates^22^ that include losses due to influenza-associated hospitalization (58%) outpatient care (4%) and mortality (38%). Clinical trial^8^ results indicate that baloxavir and oseltamivir reduce the duration of illness by at least 23 h. As the treatment rate increases, the number of courses of treatment required to avert one DALY decreases with baloxavir treatment to a greater extent than with oseltamivir treatment (Fig. 4c). For example, when only 20% of cases are treated, every 10.6 courses of baloxavir treatment is expected to avert one DALY, whereas 18.6 courses of oseltamivir treatment are needed to avert one DALY. Hence, each course of baloxavir or oseltamivir treatment is expected to prevent the loss of ~5 weeks or ~3 weeks of healthy life, respectively.

Proactive case identification and antiviral treatment can significantly mitigate the burden of seasonal influenza in the United States. Using an influenza transmission model fitted to a recent clinical trial and incidence reports from the 2017 to 2018 season, we find that baloxavir offers individual-level and population-level benefits to a greater extent than oseltamivir. For a reasonably attainable scenario in which only 20% of cases receive baloxavir treatment within 48 h of symptom onset, the estimated herd effect is a 25% reduction in overall incidence, corresponding to ~15 million infected cases averted in the United States, potentially saving ~4200 lives. With a higher treatment rate (50%), the expected number of cases averted increases to ~37 million, potentially saving ~10,200 lives. Our results indicate that optimal reduction of overall infection occurs when a significant number of infected cases are treated with baloxavir within 24 h after symptom onset. Consequently, efforts to accelerate the diagnosis and treatment of influenza infections with antivirals such as baloxavir, including potentially cost-saving telemedicine^23^, should have far-reaching public health benefits. We expect that ongoing COVID-19 responses will vastly expand the reach and speed of telehealth and increase public awareness of antivirals. Thus, antiviral treatment of 20–30% of infected patients may be attainable in future influenza epidemics.

Influenza A viruses also cause periodic widespread pandemics usually resulting in higher mortality rates^24^. The relative benefits of mass treatment with oseltamivir and baloxavir that we have estimated for seasonal influenza epidemics should extend to pandemics, although the herd effect would likely diminish for more rapidly spreading viruses^25–28^. Even at the higher *R*_0_ values characteristic of rapidly spreading pandemic viruses, baloxavir treatment is predicted to yield a higher herd effect than oseltamivir (Supplementary Fig. 6). Seminal studies of the mitigation of influenza pandemics suggest that oseltamivir-based interventions can only partially mitigate a pandemic, with the proportion of cases averted inversely related to the treatment rate, speed of treatment, and transmission rate of the pandemic virus^29,30^. Our new estimates of time-dependent baloxavir and oseltamivir efficacy against virus spread are qualitatively consistent with these prior studies and can be readily applied to the evaluation and updating of antiviral-based mitigation of pandemics.

The critical importance of mass treatment by effective antivirals is exemplified by the global pandemic (COVID-19) caused by a novel coronavirus SARS-CoV2. As of April 2020, COVID-19 has spread to ~200 countries, infected ~2.5 million people, and claimed the lives of more than 170,000 people^31^. No antivirals specific for COVID-19 are currently available to treat patients and mitigate the spread of this virus during the time that an effective vaccine is being developed and deployed. Our results indicate that the rapid development of an antiviral against COVID-19 that, like baloxavir, quickly and almost completely inhibits COVID-19 virus replication could vastly reduce morbidity and mortality worldwide. However, the likelihood of pre-symptomatic transmission^32^ and persistent disparities in access to healthcare may hinder the efficacy of future antiviral campaigns.

We assume that the efficacy and timing of antiviral treatment estimated from a clinical trial^8^ applies to the population as a whole, and have not modeled possible biases in the data with respect to disease severity or timing of treatment. Future epidemiological studies and clinical trials may allow us to address such biases and capture two key complexities not yet considered in our models. First, we have not considered that viral kinetics and the efficacy of treatment may substantially vary across age groups and risk groups, as demonstrated by others^33^. We expect that incorporating such heterogeneity will enhance intervention assessments and the prioritization of medical resources, but not qualitatively change the results of this analysis. Second, we do not yet model the evolution and transmission of baloxavir-resistant viruses, which may alter the population-level benefits of ramping up treatment rates. In recent clinical trials, baloxavir-resistant viruses emerged in 23% of baloxavir-treated children^34^ and 9.7% of baloxavir-treated adults^8^, and in some cases prolonged symptoms and viral shedding. Combination therapy with baloxavir and a neuraminidase inhibitor (oseltamivir) may prevent the generation of baloxavir-resistant viruses, whereas preserving the strong herd effect provided by baloxavir treatment. Clinical evaluation of this combination therapy is currently underway with results expected in March 2021 (ref. ^35^). A prior study provides a flexible framework for estimating the efficacy of combination therapy depending on the timing of administration^36^. As another caveat, we follow prior studies^14,15^ in assuming that the infectiousness of a case is logarithmically related to their viral load. Although there is little doubt that infectiousness and viral load are positively correlated, transmission also depends on contact patterns during the time that an individual is infectious^37^. We do not consider infection-mediated changes in contact rates, such as when individuals choose to stay home from school or work when ill.

In conclusion, our results indicate that both the scaling up and acceleration of baloxavir treatment would avert substantial influenza morbidity and mortality every year. Even modest baloxavir treatment rates can potentially spare millions of people from influenza virus infections during epidemics, thereby substantially reducing hospitalizations, morbidity, and deaths. This prediction provides an added incentive for accelerated healthcare delivery systems such as telemedicine and the development of rapid, sensitive assays for influenza virus infection.

## Methods

Our hierarchical method includes three steps (Supplementary Table 1): (i) fitting a within-host model of antiviral-induced inhibition of influenza virus replication to clinical trial data to estimate the impact of treatment on the infectiousness of patients (2) fitting a between-host model of person-to-person virus transmission to seasonal influenza surveillance data to estimate influenza transmission rates, and (3) incorporate both sets of estimates into our simulation model to project the impacts of expanding and accelerating antiviral treatment during emerging epidemics.

### Within-host model of influenza A replication dynamics

We applied a published model that includes viral suppression by both the immune response and antiviral treatment^10,11^, as given by dU/dt = −bUV;  dF/dt = bUV−δF; dZ/dt = rZ; dV/dt = (1−ϵ)pF-cV-kZV. The variables *U, F*, *Z*, and *V* represent the numbers of uninfected target cells, the numbers of infected target cells, the intensity of the immune response (i.e., antibody levels), and the amount of free virus (in TCID_50_/ml), respectively. The parameters *p*, *c, b*, *r*, and $\epsilon$ denote the viral replication rate, viral death rate, cell infection rate, growth rate of the immune response, and the antiviral efficacy. Using published estimates for the initial values of *F* and *U*^10^, we applied simulated annealing and approximate Bayesian computation^38,39^ to fit the model to clinical trial data^8^ to estimate all model parameters. We assumed that the time from infection to symptom onset follows a lognormal distribution, *L*, and the time from symptom onset to treatment follows a gamma distribution truncated at 48 h, *G*_0–48_ (ref. ^40^) the two distributions were estimated from data provided in refs. ^8,41^ using the interior-point method to minimize the root-mean-square error (Supplementary Fig. 2). We do not explicitly consider other sources of heterogeneity in viral replication or immune response rates. Although most of the patients in the trial were infected by influenza A viruses, ~10% were infected by influenza B viruses. When we consider the reduced efficacy of baloxavir against influenza B viruses relative to influenza A viruses^42^, the predictions are relatively unchanged (Supplementary Fig. 7). Following refs. ^14,15^, we assume that infectiousness is a logarithmic function of viral load, as given by $1 - e^{ - {\mathrm{log}}_{10}{\mathrm{V}}(t)/100}$ where *V(t)* denotes the virus load at time *t* since infection (Supplementary Section 2). To estimate total reduction in infectiousness attributable to treatment, we calculate the area between the infectiousness curves estimated for placebo and treatment throughout the entire period of viremia.

### Between-host influenza transmission models

Using approximate Bayesian computation^38,39^, we fit a deterministic compartmental susceptible-exposed-symptomatic-recovered (SEYR) model^43^ to incidence time series for the 2016–2017, 2017–2018, and 2018–2019 influenza seasons in the United States to estimate seasonal transmission parameters (Table 1 and Supplementary Table 3). Following refs. ^44,45^, flu incidence is estimated as the product of CDC-reported ILINet activity and WHO lab percent positive influenza tests^12,13^. We then incorporated viral replication dynamics and antiviral treatment into a stochastic agent-based version of the fitted SEYR model (Supplementary Section 3). We replace the discrete exposed and symptomatic states with continuously changing infectiousness from the moment of infection that is governed by our within-host model. Exposed individuals become symptomatic (and thus eligible for treatment) according to *L*; treated cases obtain their first dose within a 48 h window following distribution *G*_0–48_ (unless otherwise specified); symptomatic recover when their virus load falls below zero yielding average infectious periods of 9, 4, and 7 days, as infection for untreated, baloxavir-treated, and oseltamivir-treated cases, respectively (assuming treatment times follow *G*_0–48_). The force of infection (the probability that a susceptible individual becomes exposed) is given by $\lambda = \frac{{\mathop {\sum }\nolimits_{j \in Y\mathop { \cup }\nolimits^ T}^{} \beta _j(t)}}{N}$, where *N* is the population size and *β*_*j*_(*t*) is the transmission rate of the *j*th infectious individual (symptomatic or treated) at time *t*, which is determined by the product of a population-wide scaling factor *Φ* estimated from seasonal influenza incidence data and the individual’s infectiousness at time *t* based on the within-host viral load model. Supplementary Section 6 addresses the assumptions and robustness of the model with respect to influenza virus type.

### Estimating epidemiological quantities from simulation data

***R***_**0**_**:** To obtain the *R*_0_ of a single simulation, we calculate the average number of secondary cases produced by all individuals infected during the first week. For each scenario, we compute the mean and 95% confidence interval for *R*_0_ over 100 stochastic simulations (Supplementary Section 4).

### Treatment effects

To estimate the epidemiological benefits of various interventions, we conduct pairwise experiments in which we repeatedly run baseline and treatment simulations in tandem, assuming a total population of 10,000. For each pair *i*, we record the difference in total incidence between the baseline and treatment simulations, *d*_*i*_ = *I*_0_* − I*_*t*_, the total number of cases treated in the treatment simulation *n*_*i*_. For each treatment scenario, we report medians and other distributional statistics over 1000 pairs of simulations. To obtain the expected *number* of cases averted on a national-scale in the United States, we multiply the median value of *d*_*i*_*/I*_0_ by a CDC reported estimated for number of infections during the 2017–2018 influenza season^12,13^.

### DALYs averted

To estimate the DALYs averted by mass antiviral treatment, we again pair baseline and treatment simulations. To translate infections averted into healthy life years gained, we apply a published model^22^ that considers US age-specific risks, disability weights, and durations of clinical outcomes. To quantify the direct benefits for treated cases, we estimate the years averted owing to alleviation of influenza symptoms using baloxavir or oseltamivir^8^ (Supplementary Section 5).

### Reporting summary

Further information on research design is available in the Nature Research Reporting Summary linked to this article.

## Supplementary information

Supplementary Information

Reporting Summary

## Data Availability

The clinical trial data used is publicly available in ref. ^8^. All other data are available from the corresponding author upon reasonable requests.

## Code availability

Code developed R (Version 3.6.3) and Matlab (Matlab R2018b) for both the within-host and between-host models and for estimating epidemiological parameters are available from github (https://github.com/MeyersLabUTexas/baloxavir).
