# Supplementary material for: Modeling mitigation of influenza epidemics by baloxavir
Source: Nat Commun. 2020 Jun 2;11:2750. doi: 10.1038/s41467-020-16585-y (PMC7265527; doi:10.1038/s41467-020-16585-y)
Supplement: Supplementary file 1 — Supplementary Information [file 41467_2020_16585_MOESM1_ESM.pdf]

Potential mitigation of influenza epidemics by a novel antiviral

Supplementary Information

Table of contents

S1. Within-host model: Impact of antiviral treatment on the cell-to-cell proliferation of influenza 1

S2. Mapping viral load to infectiousness 7

S3. Between-host model: Impact of baloxavir or oseltamivir treatment on the transmission dynamics of influenza A 7

S4. Varying the basic reproduction number (R_0) 10

S5. Estimating the DALY's averted by scaling up antiviral treatment 12

S6. Influenza virus types 14

Supplementary Figures

Figure 1. Trajectory of a typical patient in the antiviral clinical trial. 2

Figure 2. Observed and predicted distributions for the length of time between infection and symptom onset and the length of time between symptom onset and the initiation of treatment for influenza A. 2

Figure 3. Model predictions of viral load depending on treatment initiation time following infection. 6

Figure 4. Estimated influenza incidence throughout three recent influenza seasons at a national-level in the US. 8

Figure 5. Estimated influenza virus incidence for an epidemic resembling the (a, b) 2016-2017 and (c, d) 2018-2019 influenza virus seasons in the US without and with oseltamivir or baloxavir treatment. 10

Figure 6. Impact of treating 50% of cases with baloxavir or oseltamivir for influenza viruses with different basic reproduction numbers (R_0). 12

Figure 7. Changes in viral load following treatment with baloxavir, assuming that 10% and 90% of cases are infected with influenza B and influenza A viruses, respectively. 14

Supplementary Tables

Table 1. Parameter estimation methods for the within-host and between-host models. 4

Table 2. Additional within-host parameter estimates. 5

Table 3. Additional between-host parameter estimates. 9

Table 4. Basic reproduction number (R_0) under various levels of baloxavir and oseltamivir coverage in a model based on the 2017-2018 influenza season. 11

S1. Within-host model: Impact of antiviral treatment on the cell-to-cell proliferation of influenza

We model viral replication dynamics within an individual infected by influenza A virus using the deterministic model[1,2] given by

$$\frac{dU}{dt} = -bUV \qquad \frac{dF}{dt} = bUV - \delta F \frac{dZ}{dt} = rZ \qquad \frac{dV}{dt} = (1-\epsilon)pF - cV - kZV$$

where U, F, Z and V track the number of susceptible target cells, infected cells, free virus and immune response, respectively. We use the subscript '0' to denote the initial values of these variables at the time of infection. Parameters $b, \delta, r, \epsilon, p, c$ and k govern the cell infection rate, infected cell death rate, immune response growth rate, antiviral efficacy, virus production rate, and unit conversion constant, respectively.

In fitting this model to the clinical trial data[3], we explicitly model several different time points for each patient: the time between infection and symptom onset, the time between symptom onset and the initiation of treatment, and the time of day at which viral load is measured in each day following treatment (Supplementary Figs. 1 and 2). First, we describe the distributions that we use to model treatment and testing times; then, we describe our method for incorporating these time points into the model when fitting it to clinical data.

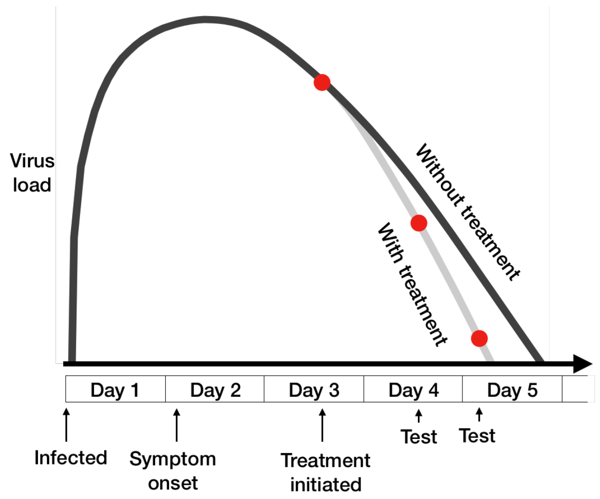

Supplementary Figure 1. Trajectory of a typical patient in the antiviral clinical trial[3]. Patients are infected at the beginning of day one, experience their first symptoms an average of 37 hours following infection (Supplementary Fig. 2a), and seek treatment an average of 23 hours after symptom onset (Supplementary Fig. 2b). Their viral load is measured only when treatment is initiated and once per day thereafter, as indicated by red points on the viral load curve.

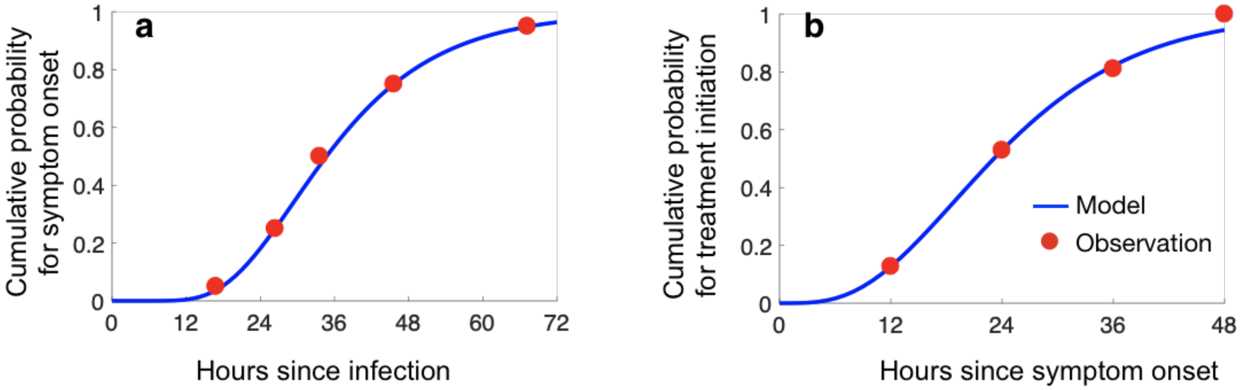

Supplementary Figure 2. Observed and predicted distributions for the length of time between infection and symptom onset and the length of time between symptom onset and the initiation of treatment for influenza A. (a) To model the timing of symptom onset (following infection) for influenza A, we fit a lognormal distribution to summary statistics provided in Table 3 of ref.4, specifically the 5th, 25th, 50th, 75th and 95th percentiles of the distribution. (b) To model the timing at which treatment is initiated following symptom onset, we fit a gamma distribution to the reported treatment times in the recent clinical trial of baloxavir and oseltamivir[3] (in 12-hour intervals). In both cases, we used the interior-point method[4] to minimize the root-mean-square error. The fitted parameters are given in Table 1.

Symptom onset time: The timing of symptom onset for influenza A virus has been shown to follow a lognormal distribution[5]. We estimated the full distribution from percentiles reported in Table 3 of Ref.[5] using an algorithm that minimizes the root-mean-square deviation between the fit model and observations (Supplementary Fig. 2a).

Treatment initiation time: To develop a plausible model for the timing of treatment following symptom onset, we analyzed data from the recent clinical trial that enrolled 1014 patients when they sought treatment for influenza-like-illness. Among the participants, 13% initiated treatment within 12 hours of symptom onset, 52% within 24 hours, 83% within 36 hours, and 100% within 48 hours[3]. We fit several different models to the data, including gamma, lognormal and Weibull distributions, using the interior-point algorithm. We chose the fitted distribution that yielded the smallest root-mean-square error between the observed and predicted values. The selected gamma distribution is given in Table 1 and shown in Supplementary Fig. 2b.

Daily viral load test time: Patients in ref.[3] were tested daily for virus load following treatment. However, the study does not report the exact time of day. We therefore assume that each round of treatment and daily testing occur sometime between 6am to 6pm, uniformly distributed.

Incorporating timing into the within-host model: We built an individual-based model that combines the deterministic viral replication dynamics with the stochastic timing of symptom onset, treatment initiation and follow-up testing. For each newly infected patient, we use the distributions described above to generate 10 timestamps: the time of symptom onset (T_S), the time of treatment initiation (T_T), and the time of eight subsequent daily viral load tests (T_{Vi}). The model then generates the patient's viral load curve using the deterministic within-host model (with the fitted parameters given in Table 1) to make hourly updates. It incorporates baloxavir or oseltamivir at the estimated efficacy if and when the patient is scheduled for treatment. The model *records* the following data for purposes of comparison to the clinical data[3]: the viral load when treatment is initiated (T_T) and at the eight daily follow-ups (T_{Vi}).

Model fitting: The within-host model described above was originally analyzed in ref.[1] with parameter values based on published studies[6–8]. Here, we fix only two parameters (I_0 and T_0) to values reported in ref.[1] and estimate all other parameters by fitting the model to the clinical data in ref.[3]. We use approximate Bayesian computation (ABC)[9] to estimate parameters where possible, and simulated annealing (SA)[10] to optimize the basic within-host parameters (Supplementary Table 1). ABC is a likelihood free method of approximating Bayesian posterior distributions that proceeds by sampling from the prior of the parameter, generating data based on the sampled parameter, and accepting or rejecting the parameter based on the distance between the generated data and the observed data. Since the distance between the generated data and the observed data is not usually a sufficient statistic, ABC produces overestimates of the credible intervals of the approximated posteriors. For a review for ABC methods please see ref.[11]. We follow the MCMC-ABC algorithm proposed by Marjoram[12] with an added modification proposed by Wilkinson[13] that includes a kernel function of the distance in the acceptance ratio. We use Euclidean distance for our distance metric, and use a normal kernel to modify our acceptance criterion.

Supplementary Table 1. Parameter estimation methods for the within-host and between-host models. We fit our models to clinical trial[3] and seasonal influenza surveillance[14,15] data using generalized simulated annealing (SA)[10], and approximate Bayesian computation (ABC)[9] as specified in the table.

**Within-host model**		
**Estimated Parameters**	**Clinical data[3]**	**Methods**
Basic within-host parameters	Placebo group	SA[10]
Antiviral efficacy for baloxavir (ϵ_B)	Baloxavir group	ABC[9]
Antiviral efficacy for oseltamivir (ϵ_O)	Oseltamivir group	ABC[9]
**Between-host model**		
**Estimated Parameters**	**Influenza surveillance data[14,15]**	**Methods**
Transmission scaling factors for the 2016-2017, 2017-2018, and 2018-2019 influenza seasons (ϕ)	2016-2017, 2017-2018, 2018-2019	ABC[9]

We use the model to simulate the clinical study of ref.[3], with 210, 427 and 377 patients receiving placebo, baloxavir and oseltamivir, respectively. For each patient in each group, we simulate their viral load throughout the course of their infection (i.e., from initial infection through nine days after treatment), but record only the viral load at the time that treatment is initiated and at each daily follow-up test. We aggregate the simulated data by treatment group and calculate the mean and standard deviation in change in viral load as a function of days since the initiation of treatment.

We divide the parameters into the following two groups and take a two-step approach to their estimation. First we use SA to stochastically search the parameter space of basic within-host viral growth parameters (cell infection rate b, virus production rate p, initial sensitive viral load V_0, virus death rate c, initial immune response Z_0, infected cell death rate δ, immune response growth rate r) and identify the optimal parameters based on the MSE between the observed viral load change from the placebo group data in ref.[3] and the estimated viral load change. We then fix these parameters at their optimal values and estimate the antiviral efficacies (ϵ) for baloxavir and oseltamivir by fitting to the corresponding clinical trial data[3] using approximate Bayesian computation (ABC) with 10,000 iterations after burn-in (9,000)[16].

Approximately 90% of patients in the focal clinical trial[3] were infected by influenza A H3N2, which was the dominant seasonal influenza virus during the 2016-2017 and 2017-2018 US seasons. Therefore, the fitted within-host model is primarily informed by influenza A H3N2. Although only 1.5% of the trial patients were infected by influenza A H1N1, we expect that the estimated baloxavir and oseltamivir efficacies will be reasonable approximations for all influenza A viruses (H3N2 and H1N1), as suggested by refs.[17,18]. The within-host model parameters have previously been estimated for influenza A H1N1 but not influenza A H3N2[1]. Thus, we constrained the prior parameter distributions using both the prior H1N1 estimates[1] and a published study suggesting that H3N2 has a higher virus production rate p and initial sensitive viral load V_0 than influenza A

H1N1[19] (differing by less than a factor of 10^4), while the other viral growth parameters are quite similar (differing by less than a factor of 10). In the ABC for the efficacy of baloxivir and oseltamivir we assume a U(0,1) prior for each. For the optimization of within-host parameters, we specify the following restrictions on our parameter space.

- p and V_0: between 1 and 10^4 times the published values of 1.2×10^{-5} $TCID_{50}$/ml per day and 0.0077 $TCID_{50}$/ml for influenza A H1N1[1]
- b, c, Z_0 and r: between 0.1 to 10 times the published values of 0.099 ml/$TCID_{50}$ per day, 0.081 $days^{-1}$, 0.34, and 1 $days^{-1}$ for influenza A H1N1[1]

In addition, the clinical trial[3] focused on the *change in viral load* between successive daily measurements. The observed standard deviation in this value across patients consistently exceeded 1.4 (log_{10} $TCID_{50}$/ml). To constrain our model accordingly, we disallow parameter combinations that predict low standard deviation in daily change in viral load below a certain threshold. To optimize the within-host parameters, we run the SA algorithm 300 times with different starting points. We use the median values of the parameters from these 300 runs as our estimate. To give an idea of the ranges of our estimates we provide the 2.5th and 97.5th percentiles for each of the parameters from the output of the 300 runs. We then fix these parameters at their median values, and apply ABC[16] to estimate the efficacies of baloxivir and oseltamivir from the treatment group data[3]. The estimates are provided in Table 1 and Supplementary Table 2.

Supplementary Table 2. Additional within-host parameter estimates. These parameters are estimated by fitting the within-host model to clinical trial data for the placebo treatment group[3] using simulated annealing algorithms[10] with 300 different starting points.

**Fixed Parameter**	**Value**	**Reference**	
Initial number of infected cells (F_0)	0	Ref. [1]	
Initial number of target cells (U_0)	4×10^8	Ref. [1]	
**Estimated Parameter**	**Median**	**2.5% Percentile**	**97.5% Percentile**
Cell infection rate in ml/$TCID_{50}$ per day (b)	0.26	0.10	0.71
Virus production rate in $TCID_{50}$/ ml per day (p)	0.04	0.03	0.10
Virus death rate in $days^{-1}$ (c)	0.27	0.02	0.56
Initial immune response (Z_0)	0.05	0.04	2.21
Infected cell death rate in $days^{-1}$ (δ)	1.68	1.49	1.92
Immune response growth rate in $days^{-1}$ (r)	2.34	1.47	2.60

The viral load predictions of the fitted model at the time of treatment are consistent with the clinical data (Fig. 1). The model estimates the average (±standard deviation) viral loads at the time of

treatment as 5.51±1.67, 5.48 ±1.65 and 5.41±1.67 $\log_{10}$ $TCID_{50}$/ml among patients treated with baloxavir, oseltamivir and placebo, respectively, which are consistent with the observed values of 5.79±1.87, 5.94±1.69 and 5.56±1.89 $\log_{10}$ $TCID_{50}$/ml[3], respectively.

In summary, we analyzed antiviral clinical trial data[3] that are provided as aggregated measurements of changes in viral load (TCID50/ml) following treatment (i.e., mean and standard deviation across patients), rather than measurements of absolute viral load in individual patients. The fitted model allows us to estimate a key input of our population-level influenza transmission model–the viral load of individual cases with or without treatment as a function of the lag between symptom onset and antiviral administration. For example, Supplementary Fig. 3 shows the estimated viral loads of patients receiving treatment at four different intervals following symptom onset, based on the fitted parameters.

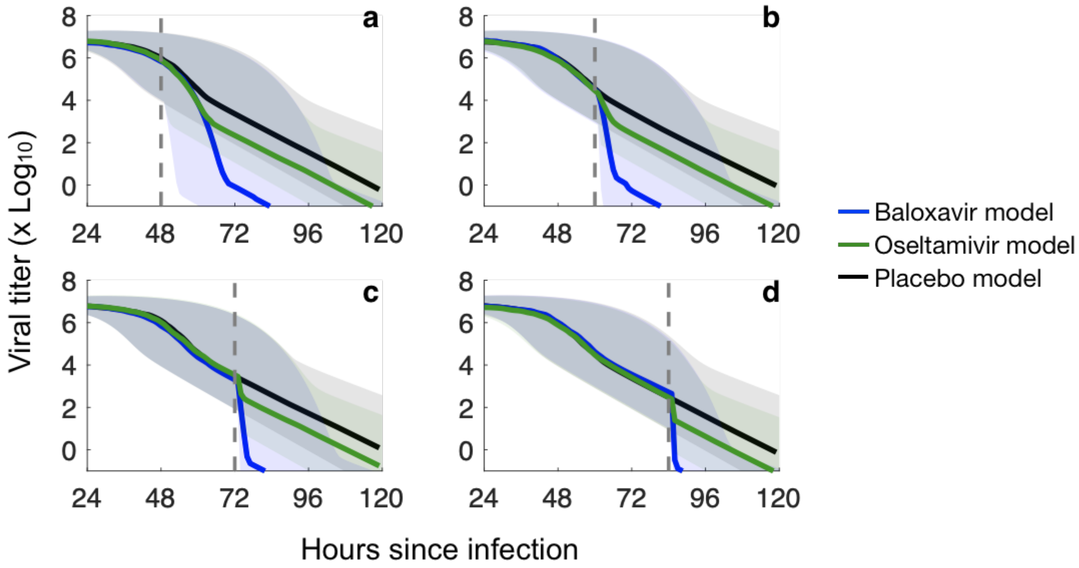

Supplementary Figure 3. Model predictions of viral load depending on treatment initiation time following infection. Using the parameters fit to clinical trial data[3] (Table 1 and Supplementary Table 2), we use our model to estimate the changing viral load within individual patients following treatment at four different treatment times (dashed lines): (a) 12 hours, (b) 24 hours, (c) 36 hours, and (d) 48 hours after symptom onset. We assume a 37 hour lag from infection to symptom onset, based on the mean of lognormal distribution given in Table 1. Lines indicate medians; shading indicates the full range of values produced by 100 stochastic simulations, each with parameters randomly sampled from 95% ranges given in Table 1 and Supplementary Table 2.

S2. Mapping viral load to infectiousness

To evaluate the impact of antiviral therapy on the transmission dynamics of influenza, we model the relationship between the viral load and infectiousness of an individual. Prior studies have explored three types of viral load-infectiousness coupling functions: logarithmic,

sigmoid and linear[20]. Following other influenza modeling studies[21,22], we adopt a logarithmic relationship between infectiousness and viral titer (which requires fewer parameters than a sigmoidal function). Specifically, we use a functional form suggested in ref.[23]: the infectiousness of individual j at time t is given by $\beta_j(t) = \phi\iota_j(t)$ where ϕ is the population baseline transmission rate estimated from seasonal influenza incidence data and $\iota_j(t) = 1 - e^{-log_{10}V_j(t)/100}$ is an infectiousness scaling factor with $V_j(t)$ denoting the viral titer of j at time t.

S3. Between-host model: Impact of baloxavir or oseltamivir treatment on the transmission dynamics of influenza A

We analyze both a deterministic model and an individual-based model of influenza transmission in the US. Our deterministic susceptible-exposed-symptomatic-recovered (SEYR) model is standard (as in refs.[24–26]) and does not incorporate antiviral therapy. We use ABC[16] to fit it to seasonal influenza data to estimate baseline transmission parameters for the 2016-2017, 2017-2018, and 2018-2019 influenza seasons in the US.

Our stochastic individual-based model tracks the progression of cases through the following states: susceptible (S), exposed (E), symptomatic (Y), treated (T) and recovered (R). Susceptible individuals become infected (move from S to E) at a rate of λ, given by

$$\lambda = \frac{\sum_{j \in Y \cup T} \quad \beta_j(t)}{N}$$

where N is the total population size and $\beta_j(t)$ is the transmission rate of the jth infectious individual at time t. We assume that $\beta_j(t)$ is the product of a population-wide scaling factor (ϕ) estimated from seasonal influenza incidence data and individual j's viral load at time t. We update the model once per day, with the infectiousness of each case assumed to equal the average over the preceding 24 hours.

Once an individual is infected, we use the within-host model to explicitly track their changing viral load. They progress to symptomatic following the fitted lognormal distribution L of symptom onset (Table 1). They are randomly selected for treatment according to the specified case treatment rate (which is zero for baseline simulations without intervention). If treated, the timing of treatment after symptom onset is randomly generated from the specified distribution of treatment times. Unless otherwise specified, we assume that treatment is initiated within the first 48 hours of symptom onset according to the distribution estimated from ref.[3] (Table 1). The individual recovers when their viral load falls below zero.

We denote the proportion of susceptible individuals in the population prior to the epidemic as $p_{\text{susceptible}}$ and the initial proportion of population infected as p_{I_0}. We assume that $p_{\text{susceptible}}$ is the proportion of the population that were not effectively vaccinated for the current season. This ignores individuals who were naturally immunized by prior influenza infections. Following ref.[27,28], we assume that the proportion of the population that is immunized is given by $\kappa\omega$, where κ denotes vaccine efficacy and ω is the vaccination coverage at the population level. Accordingly, we set $p_{\text{susceptible}} = 1 - \kappa\omega$. The CDC published estimates for κ and ω of 0.40 and 0.398 in the 2016-2017 season, 0.40 and 0.386 in the 2017-2018 season, and 0.47 and 0.449 in the 2018-2019 season, respectively[14,15]. For all models, we set p_{I_0} equal to 0.01, which is consistent with the reported flu

incidence (the product of CDC-reported ILINet activity and WHO lab percent positive influenza tests[14,15]) in the starting week of each season.

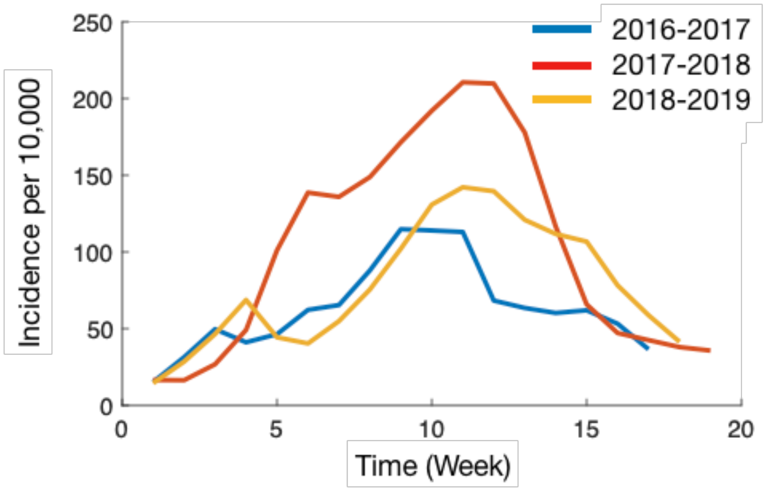

Supplementary Figure 4. Estimated influenza incidence throughout three recent influenza seasons at a national-level in the US. Values are the product of CDC ILINet estimates of influenza activity (proportion of clinical visits attributed to influenza-like-illness)[14] and WHO Public Health Laboratory estimates of percent positive flu tests[15], as suggested by ref.[30].

Given that disease progression is governed by the fitted within-host model, the only remaining parameter is the transmission scaling factor (ϕ) that may differ across epidemics. For each of the three focal influenza seasons (i.e., 2016-1017, 2017-2018, and 2018-2019), we estimate ϕ by fitting the stochastic individual-based SEYR model (without treatment) model to an influenza incidence time series estimated from national-scale US ILI and laboratory surveillance data (Supplementary Fig. 4) using an ABC algorithm. We first run 100 stochastic simulations of the SEYR model, and for each of these we simulate 1,000 draws from the posterior of ϕ (after discarding the first 9,000) using ABC. We proceed using the same ABC algorithm outlined for the efficacies in the within-host model, and assume a uniform (0,1) prior for ϕ. The combined draws across all SEYR models constitute our estimate of ϕ. Table 1 and Supplementary Table 3 provide the fixed and estimated parameters for the three influenza seasons. Supplementary Fig. 5 provides results based on parameters fitted to the epidemic curves for the 2016-2017 and 2018-2019 influenza seasons.

Since baloxavir is not yet approved for children in the US (as it has in Japan), age-specific estimates would be hypothetical at this point, but quite valuable once data are available. Incorporating such complexities will ultimately increase accuracy, but will not likely change the qualitative results of this study[29].

Supplementary Table 3. Additional between-host parameter estimates. Epidemiological parameters are fixed with respect to public health surveillance reports[14,15] or estimated by approximate Bayesian computation[9,11] with 10,000 iterations after burn-in (9,000).

**Fixed Parameter**	**Value**	**Reference**

Initial proportion of population susceptible to influenza prior to the 2016-2017 season	84.08%	refs.[27,28]	
Initial proportion of population susceptible to influenza prior to the 2017-2018 season	84.56%	refs. [27,28]	
Initial proportion of population susceptible to influenza prior to the 2018-2019 season	78.90%	refs.[27,28]	
Initial proportion of infectious people p_{I_0} in flu seasons	0.01%		
**Estimated Parameter**	**Median**	**Lower 95% CI**	**Upper 95% CI**
Transmission scaling factor (ϕ) estimated from 2016-2017 seasonal influenza data	2.79	2.77	2.80
Transmission scaling factor (ϕ) estimated from 2017-2018 seasonal influenza data	2.91	2.86	2.93
Transmission scaling factor (ϕ) estimated from 2018-2019 seasonal influenza data	3.01	3.00	3.03
Transmission scaling factors (ϕ) for R_0 = 1.2, 1.3, 1.4, 1.5, 1.6 and 1.7 scenarios	3.03, 3.24, 3.56, 3.83, 4.08, 4.38		

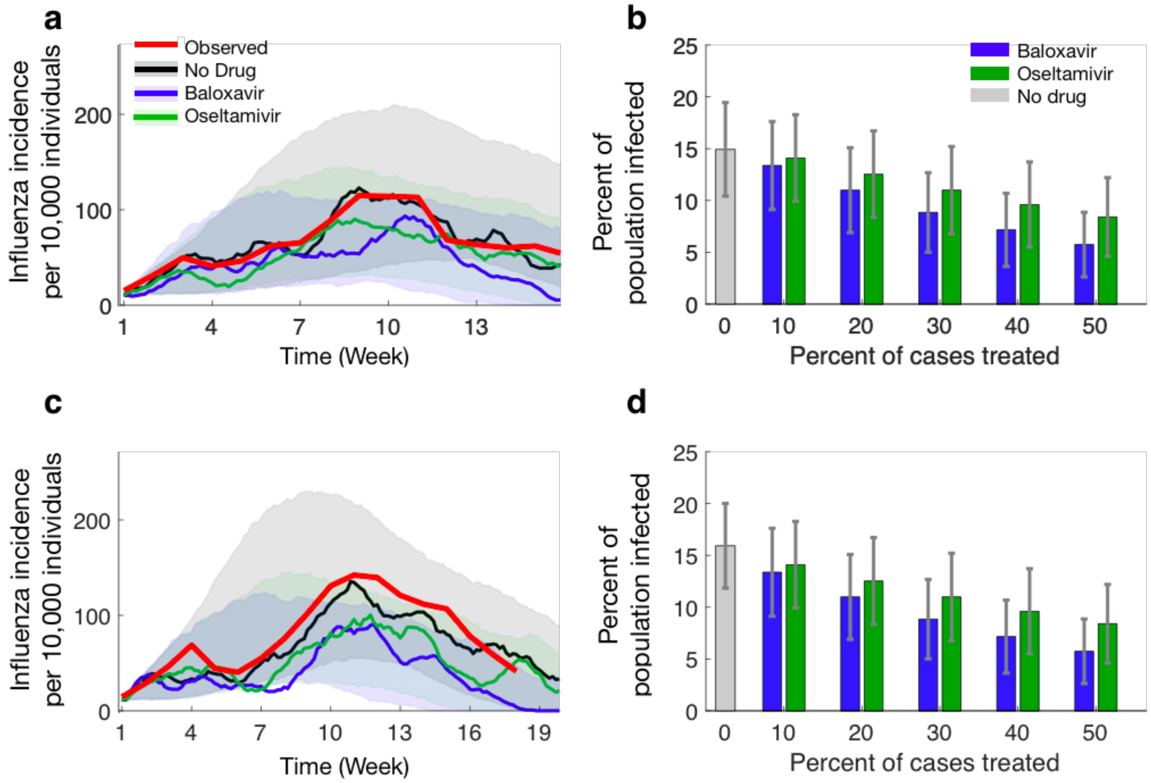

Supplementary Figure 5. Estimated influenza virus incidence for an epidemic resembling the (a, b) 2016-2017 and (c, d) 2018-2019 influenza virus seasons in the US without and with oseltamivir or baloxavir treatment. (a, c) Observed incidence of influenza virus (red) based on US surveillance data compared to typical model simulations without any antiviral treatment (black) or with 30% of cases receiving baloxavir (blue) or oseltamivir (green) treatment. Lines indicate a moving ten-day average of incidence; shading corresponds to the middle 80% of values across *n*=1,000 independent stochastic simulations. (b, d) Estimated total attack rates in simulated intervention scenarios ranging from no cases receiving antiviral treatment to 50% of cases treated. The heights of the columns and error bars show the median values and interquartile range, respectively, across *n*=1,000 independent stochastic simulations for each scenario. Each stochastic simulation assumes a population of 10,000 individuals, with within-host viral replication and between-host transmission parameters given in Table 1 and Supplementary Tables 2 and 3.

S4. Varying the basic reproduction number (R_0)

To estimate R_0 from a simulated epidemic, we simply calculate the average number of secondary cases produced by individuals infected during the first. For each scenario, we run 100 stochastic simulations and report the mean and 95% confidence interval in R_0 (Supplementary Table 4).

To model viruses with higher R_0 than observed during 2017-2018 influenza season, we increased the transmission scaling factor (ϕ) from its baseline estimate in increments of 0.01 and estimated the resulting mean R_0 across 100 stochastic simulations. For each desired R_0 (i.e., 1.2, 1.3, 1.4, 1.5, 1.6, 1.7), we choose the ϕ that minimized the root-mean-square error between the target value and

the simulated mean R_0 (Supplementary Table 4).

To assess the sensitivity of our results to the basic reproduction number, we simulated a 50% treatment rate for each of the drugs (Supplementary Fig. 6).

Supplementary Table 4. Basic reproduction number (R_0) under various levels of baloxavir and oseltamivir coverage in a model based on the 2017-2018 influenza season.

Percent of cases treated	Baloxavir			Oseltamivir		
	Mean	95% CI lower	95% CI upper	Mean	95% CI lower	95% CI upper
0	1.15	1.12	1.17	1.15	1.12	1.17
10	1.12	1.09	1.14	1.13	1.10	1.15
20	1.09	1.07	1.12	1.11	1.08	1.13
30	1.08	1.05	1.10	1.09	1.07	1.12
40	1.06	1.04	1.08	1.09	1.06	1.11
50	1.04	1.02	1.06	1.07	1.05	1.10

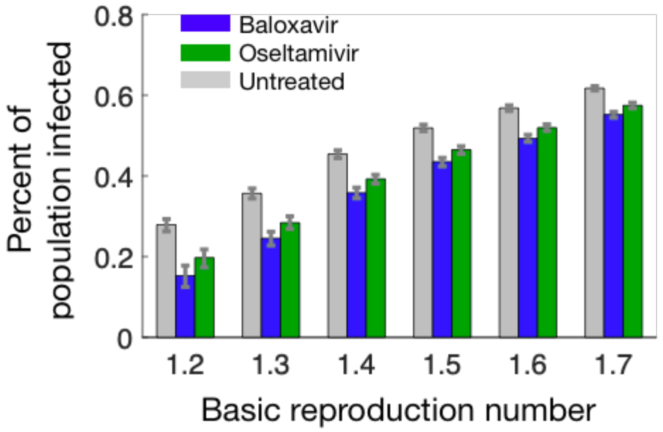

Supplementary Figure 6. Impact of treating 50% of cases with baloxavir or oseltamivir for influenza viruses with different basic reproduction numbers (R_0). To model viruses with higher reproduction numbers, we increase the scaling factor ϕ in our fitted 2017-2018 seasonal influenza model accordingly (Supplementary Table 3). The baloxavir (blue) and oseltamivir (green) scenarios assume that 50% of cases are treated and that each course of treatment is initiated within 48 hours of symptom onset. The heights and error bars indicate medians and interquartile ranges across n=1,000 independent stochastic simulations, each of which assumes a population of 10,000 individuals, with within-host viral replication and between-host transmission parameters given in Table 1 and Supplementary Tables 2 and 3.

S5. Estimating the DALY's averted by scaling up antiviral treatment

For each antiviral scenario, we run 1000 pairs of baseline and treatment simulations in which all settings are identical except for those governing antiviral treatment. For each pair, we determine the total incidence produced in the baseline (I_0) and treatment (I_t) and calculate $(I_0-I_t)/I_0$ as an estimate of the proportion of infections averted by the antiviral intervention. We also record the proportion of the population that were administered antivirals in the treatment simulation. To obtain the expected *number* of cases averted on a national-scale in the US, we multiply the median proportion of infections averted by a CDC reported estimated for number of infections during the 2017-2018 influenza season[14,15].

The Disability-Adjusted Life Year (*DALY*) is a measure of overall disease burden in terms of lost years of healthy life due to infection. We run pairs of baseline and intervention simulations and calculate the resulting difference in DALY's. Each estimate considers both the direct benefit of treatment (reduced duration of symptoms for treated cases) and the indirect population-wide benefits achieved by preventing infections. Both types of benefits are quantified in terms of the prevention of influenza-associated years lost due to disability (*YLD*) or life loss (*YLL*). For each pair of simulations we estimate the DALY's averted as a sum of three separate quantities (C_l, C_d and C_t), as follows:

1. For each pair of simulations, we calculate the difference in overall incidence $\Delta = I_0 - I_t$. We then estimate the *age-specific* reduction in overall incidence as $\Delta_a = \Delta \cdot \rho_a$ where ρ_a is the proportion of infections occurring among individuals that are a years old. We estimate ρ_a from published estimates of age-specific burden of influenza for each focal influenza

season[14,15]. We make the simplifying assumption that infections are distributed uniformly across each year in a given age group. For example, during the 2017-2018 influenza season, an estimated 22% of infections occurred in 0-4 year olds; we assume that 4.4% of infections occurred in each of five constituent single-year groups (i.e., $\rho_0 = \rho_1 = \rho_2 = \rho_3 = \rho_4 = 0.044$).

2. We estimate the YLL prevented by the intervention as

$$C_l = \sum_a \Delta_a(\lambda_a - a)\delta_a$$

where λ_a denotes the life expectancy for individuals of age a (obtained from ref. [31]) and δ_a denotes the age-specific case fatality rate for seasonal influenza (obtained from ref. [32]).

3. We use published estimates of age-specific disabilities attributable to influenza infections[27] to calculate the YLD prevented by the intervention. Ref.[27] considers six different categories of clinical severity, ranging from uncomplicated outpatient cases to complicated hospitalized cases including pneumonia. It provides estimates of (i) the probability that an infected individual of age a will fall into severity category C ($\zeta_{a,C}$), (ii) the per-day disability weight for an infected individual in severity category c (w_C), and (iii) the duration of disability for an individual of age a in category C, which is equal to either a specified number of weeks, the infectious period, or the remaining life expectancy ($\delta_{a,C}$). We use these values (Table S4 from ref.[27]) to calculate

$$C_d = \sum_a \sum_C w_c[\Delta_a \zeta_{a,C} \delta_{a,C}].$$

4. We estimate the direct benefits of antivirals to treated patients as

$$C_t = \tau \cdot v_i$$

where τ is the number of cases that received treatment in the intervention simulation and v_i is reduction in years of life lost due to disability achieved by a single course of treatment with drug i. The recent clinical trial[3] reports that baloxavir and oseltamivir accelerate the alleviation of symptoms, and provides multiple estimates of the median difference in time to alleviation of symptoms (between treatment versus placebo groups), ranging from 23.2 to 38.6 hours. We conservatively assume a low estimate of 23 hours and that there is no difference between baloxavir and oseltamivir. Accordingly, the reduced YLD attributable to a single course of baloxavir or oseltamivir treatment is given by $v_B = v_O = 23/(24 \cdot 365) = .0026$ years.

For a given pair of simulations, we calculate the number of cases treated over the DALY's averted (i.e., $\tau / (C_l + C_d + C_t)$). For each intervention scenario, Fig. 4c indicates the distribution of these ratios across 1000 pairs of simulations.

S6. Influenza virus types

We fit our within-host model of viral replication to clinical trial data in which approximately 90% of the 1014 patients were infected by influenza A (H3N2) viruses and the remaining 10% were infected by influenza B viruses[3]. Thus, the composite estimates of antiviral efficacy reflect the composition of viral types circulating during the trial and the differential efficacies of the drugs against influenza A and B viruses. Our main results are based on this average efficacy.

To verify the robustness of the model, we ran an additional set of within-host simulations that explicitly incorporate the 90-10 mixed composition of viruses reported in the clinical trial. We assume that the efficacy of baloxiavir for treating influenza B is reduced by 90% relative to its efficacy for influenza A. The resulting curves (change in viral load since treatment) are similar to both the empirical data and the model predictions when we do not specify influenza type (Supplementary Fig. 7).

Although our estimates of baloxavir- and oseltamivir-mediated reductions in influenza infectiousness are not specific to viral type, we conjecture that they provide reasonable estimates for seasons dominated by influenza A viruses, such as 2016-2017, 2017-2018, and 2018-2019 during which an estimated 77%, 71% and 95% of infections were caused by influenza A viruses, respectively[34].

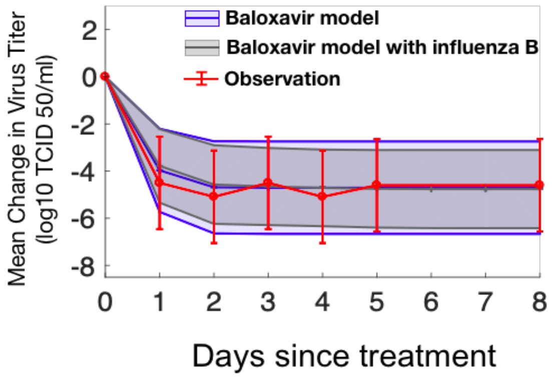

Supplementary Figure 7. Changes in viral load following treatment with baloxavir, assuming that 10% and 90% of cases are infected with influenza B and influenza A viruses, respectively. We assume that the efficacy of baloxavir against influenza B viruses is one-tenth the value estimated for influenza A viruses (Table 1). The graph compares the change in virus titer predicted by the fitted within-host model, with (blue) and without (gray) the assumption that 10% of the 1014 infections are caused by influenza B. The projections are similar to each other and closely track the empirical observations[3] among patients treated with baloxavir (427 patients). The error bars and shaded areas reflect the observed and predicted standard deviations, respectively. Day zero corresponds to the time of baloxavir administration.

Bibliography

1. Handel, A., Longini, I. M. & Antia, R. Neuraminidase inhibitor resistance in influenza: assessing the danger of its generation and spread. *PLoS Comput. Biol.* **3**, e240 (2007).
2. Smith, A. M. & Perelson, A. S. Influenza A virus infection kinetics: quantitative data and models. *Wiley Interdiscip. Rev. Syst. Biol. Med.* **3**, 429–445 (2011).
3. Hayden, F. G. *et al.* Baloxavir marboxil for uncomplicated influenza in adults and adolescents. *N. Engl. J. Med.* **379**, 913–923 (2018).
4. Nesterov, Y. & Nemirovskii, A. *Interior-point Polynomial Algorithms in Convex Programming*. (SIAM, 1994).
5. Lessler, J. *et al.* Incubation periods of acute respiratory viral infections: a systematic review. *Lancet Infect. Dis.* **9**, 291–300 (2009).
6. Baccam, P., Beauchemin, C., Macken, C. A., Hayden, F. G. & Perelson, A. S. Kinetics of influenza A virus infection in humans. *J. Virol.* **80**, 7590–7599 (2006).
7. Kiso, M. *et al.* Resistant influenza A viruses in children treated with oseltamivir: descriptive study. *Lancet* **364**, 759–765 (2004).
8. Zürcher, T. *et al.* Mutations conferring zanamivir resistance in human influenza virus N2 neuraminidases compromise virus fitness and are not stably maintained in vitro. *J. Antimicrob. Chemother.* **58**, 723–732 (2006).
9. Tavaré, S., Balding, D. J., Griffiths, R. C. & Donnelly, P. Inferring coalescence times from DNA sequence data. *Genetics* **145**, 505–518 (1997).
10. Xiang, Y., Gubian, S., Suomela, B. & Hoeng, J. Generalized Simulated Annealing for Global Optimization: The GenSA Package. *R J.* **5**, (2013).
11. Marin, J.-M., Pudlo, P., Robert, C. P. & Ryder, R. J. Approximate Bayesian computational methods. *Stat. Comput.* **22**, 1167–1180 (2012).

12. Marjoram, P., Molitor, J., Plagnol, V. & Tavare, S. Markov chain Monte Carlo without likelihoods. *Proc. Natl. Acad. Sci. U. S. A.* **100**, 15324–15328 (2003).

13. Wilkinson, R. D. Approximate Bayesian computation (ABC) gives exact results under the assumption of model error. *Stat. Appl. Genet. Mol. Biol.* **12**, 129–141 (2013).

14. Centers for Disease Control and Prevention. Influenza Positive Tests Reported to CDC by Public Health Laboratories and ILI Activity, National Summary. https://gis.cdc.gov/grasp/fluview/fluportaldashboard.html (2018).

15. Centers for Disease Control and Prevention. Percentage of visits for ILI, National Summary. https://gis.cdc.gov/grasp/fluview/fluportaldashboard.html (2018).

16. Vihola, M. Robust adaptive Metropolis algorithm with coerced acceptance rate. *Stat. Comput.* **22**, 997–1008 (2012).

17. Takashita, E. *et al.* Susceptibility of Influenza Viruses to the Novel Cap-Dependent Endonuclease Inhibitor Baloxavir Marboxil. *Front. Microbiol.* **9**, 3026 (2018).

18. Koszalka, P., Tilmanis, D., Roe, M., Vijaykrishna, D. & Hurt, A. C. Baloxavir marboxil susceptibility of influenza viruses from the Asia-Pacific, 2012–2018. *Antiviral Res.* **164**, 91–96 (2019).

19. Holder, B. P. & Beauchemin, C. A. A. Exploring the effect of biological delays in kinetic models of influenza within a host or cell culture. *BMC Public Health* **11 Suppl 1**, S10 (2011).

20. Handel, A. & Rohani, P. Crossing the scale from within-host infection dynamics to between-host transmission fitness: a discussion of current assumptions and knowledge. *Philos. Trans. R. Soc. Lond. B Biol. Sci.* **370**, (2015).

21. Tsang, T. K. *et al.* Influenza A Virus Shedding and Infectivity in Households. *J. Infect. Dis.* **212**, 1420–1428 (2015).

22. Handel, A., Brown, J., Stallknecht, D. & Rohani, P. A multi-scale analysis of influenza A virus fitness trade-offs due to temperature-dependent virus persistence. *PLoS Comput. Biol.* **9**, e1002989 (2013).

23. Handel, A., Lebarbenchon, C., Stallknecht, D. & Rohani, P. Trade-offs between and within scales: environmental persistence and within-host fitness of avian influenza viruses. *Proc. Biol. Sci.* **281**, (2014).

24. Fox, S. J., Miller, J. C. & Meyers, L. A. Seasonality in risk of pandemic influenza emergence. *PLoS Comput. Biol.* **13**, e1005749 (2017).

25. Mills, C. E., Robins, J. M. & Lipsitch, M. Transmissibility of 1918 pandemic influenza. *Nature* **432**, 904–906 (2004).

26. Flahault, A., Vergu, E., Coudeville, L. & Grais, R. F. Strategies for containing a global influenza pandemic. *Vaccine* **24**, 6751–6755 (2006).

27. Sah, P., Medlock, J., Fitzpatrick, M. C., Singer, B. H. & Galvani, A. P. Optimizing the impact of low-efficacy influenza vaccines. *Proc. Natl. Acad. Sci. U. S. A.* **115**, 5151–5156 (2018).

28. Medlock, J. & Galvani, A. P. Optimizing influenza vaccine distribution. *Science* **325**, 1705–1708 (2009).

29. Volz, E. M., Miller, J. C., Galvani, A. & Meyers, L. A. Effects of Heterogeneous and Clustered Contact Patterns on Infectious Disease Dynamics. *PLoS Computational Biology* vol. 7 e1002042 (2011).

30. Goldstein, E., Viboud, C., Charu, V. & Lipsitch, M. Improving the estimation of influenza-related mortality over a seasonal baseline. *Epidemiology* **23**, 829–838 (2012).

31. Arias, E., Heron, M. & Xu, J. United States Life Tables, 2014. *Natl. Vital Stat. Rep.* **66**, 1–64 (2017).

32. Monto, A. S. The risk of seasonal and pandemic influenza: prospects for control. *Clin. Infect. Dis.* **48 Suppl 1**, S20–5 (2009).

33. Koszalka, P., Tilmanis, D., Roe, M., Vijaykrishna, D. & Hurt, A. Baloxavir Marboxil Susceptibility of Influenza Viruses from the Asia-Pacific, 2012-2018. doi:10.1101/498766.

34. Centers for Disease Control and Prevention. National, Regional, and State Level Outpatient Illness and Viral Surveillance. https://gis.cdc.gov/grasp/fluview/fluportaldashboard.html (2018).
